# Immunoregulatory molecules secreted by *Trichuris muris*

**DOI:** 10.1017/S0031182021000846

**Published:** 2021-12

**Authors:** Allison J. Bancroft, Richard K. Grencis

**Affiliations:** 1 Lydia Becker Institute for Immunology and Inflammation, Manchester M13 9PT, UK; 2 Wellcome Trust Centre for Cell Matrix Research, Manchester M13 9PT, UK; 3 Division of Infection, Immunity and Respiratory Medicine, Manchester M13 9PT, UK; 4 School of Biological Sciences, Faculty of Biology, Medicine and Health, Manchester Academic Health Science Centre, University of Manchester, Manchester M13 9PL, UK

**Keywords:** Immunoregulation, *Trichuris*, whipworm

## Abstract

*Trichuris*, whipworm nematode infections are prevalent in humans, domestic livestock and mammals. All share an epithelial dwelling niche and similar life cycle with the chronic infections that follow implying that immune evasion mechanisms are operating. Nematode excretory secretory (ES) products have been shown to be a rich source of immunomodulatory molecules for many species. The *Trichuris muris* model is a natural parasite of mice and has been used extensively to study host–parasite interactions and provides a tractable platform for investigation of the immunoregulatory capacity of whipworm ES. The present review details progress in identification of the composition of *T. muris* ES, immunomodulatory components and their potential mechanisms of action. The adult *T. muris* secretome is dominated by one protein with modulatory capacity although remains to be completely characterized. In addition, the secretome contains multiple other proteins and small molecules that have immunomodulatory potential, certainly by comparison to other *Trichuris* species. Moreover, *T. muris*-derived exosomes/exosome-like vesicles contain both protein and multiple miRNAs providing an alternate delivery process for molecules with the potential to modulate host immunity.

## Graphical abstract

**Figure:**
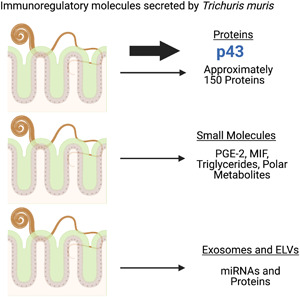

## Introduction

### The mouse model of human disease

*Trichuris* is a genus comprising intestinal nematodes including the human whipworm parasite, *Trichuris trichiura*, that is currently believed to infect approximately 465 million people worldwide causing considerable morbidity and economic hardship (Hotez *et al*., 2014; Pullan *et al*., 2014). It was estimated to be responsible for the loss of 0.64 million disability adjusted life years from 1990 to 2010 and was ranked 10th in the neglected tropical disease rankings (Hotez *et al*., 2014) with long lived, chronic infections the norm. It is mostly children who suffer pathology with *Trichuris* dysentery syndrome, growth retardation, cognitive problems and finger clubbing commonly reported (Cooper *et al*., 1991; MacDonald *et al*., 1991; Nokes and Bundy, 1992). *Trichuris trichiura* has been shown to be relatively resistant to drug treatment and reinfection after worm clearance is common (Bundy *et al*., 1987) necessitating development of new chemotherapeutics or vaccine development, both of which have proved slow. This results in a situation in which populations in endemic areas have low level worm burdens as they are constantly exposed to infectious eggs; they do however, generate partially acquired immunity with age (Bundy *et al*., 1988). Chronic human helminth infection has been associated with immunoregulation (van Riet *et al*., 2007; Smits *et al*., 2010; Alcantara-Neves *et al*., 2017) although it is important to recognize that these infections cause a considerable collective damage to endemic communities (Cooper and Bundy, 1988).

Our laboratory and many others have used the murine parasite *Trichuris muris*, a natural parasite of mice which is closely related to the human parasite to study host whipworm interactions (Cliffe and Grencis, 2004). In this scenario, many variables such as infection levels, host genetics and nutrition, can be controlled. A single low dose infection or more realistically repeated low level i.e. trickle infections (Bancroft *et al*., 2001; Glover *et al*., 2019) of *T. muris* can be used to reflect the infection levels and dynamics seen in the wild. Parasites reach patency around 33 days after infection and adult worms survive for extended periods with some adults dying from senescence from 100 days onwards.

The majority of inbred laboratory mouse strains expel a high dose infection, whereas immune-deficient mouse strains will take high dose infections through to chronicity showing that host protective immunity can operate (Cliffe and Grencis, 2004; Klementowicz *et al*., 2012; Grencis *et al*., 2014). Moreover, transient immunosuppression given to immunocompetent strains of mouse during the second week of infection will also allow progression to patency (Lee and Wakelin, 1983). Low dose and trickle infections lead to chronicity without external immunosuppression and all without obvious negative effects on the health of the host. Taken together with the observations that chronic infection is associated with qualitative and quantitative differences in the immune response generated when compared to resistant mice suggest that the parasite has immunomodulatory activity (Klementowicz *et al*., 2012; Grencis *et al*., 2014; Colombo and Grencis, 2020). This is further emphasized by blocking the regulatory cytokine, interleukin (IL)-10 during the chronic phases of infection, which leads to excessive intestinal pathology sometimes with fatal consequences (Grencis *et al*., 2014). An earlier study examined the effect of abbreviated infections or chronic infection upon the capacity of the host to clear a subsequent infection. These data strongly indicated that exposure to later stages of the infection prior to a challenge was less effective at inducing protection and thus potentially immunomodulatory (Else *et al*., 1989). Research from our laboratory using models of chemical contact hypersensitivity that depend upon type 1 or type 2 cytokine responses showed that a chronic *T. muris* infection modulated challenge responses to the sensitizers in the ear, a remote non-mucosal site distant from the caecum where the parasite resides (Grencis *et al*., 2014). It was also apparent that modulation operated only against type 1 sensitization and not type 2 sensitization influencing local cytokine responses and ear pathology. This depression in sensitization was associated with a reduction in movement of class-II positive cutaneous dendritic cells from the skin and elevated IL-10 levels in the ear draining lymph node (Grencis *et al*., 2014). This would suggest that the immunoregulation induced by *T. muris* infection operating systemically reflects that generated during chronic *T. muris* intestinal infection, i.e. a regulated Th1 response. It has also been shown that a chronic *T. muris* infection can modulate the brain inflammatory response in stroke (Denes *et al*., 2010). Systemic effects of chronic *T. muris* infection were subsequently observed by Chenery *et al*. (2016) where haematopoetic responses in the bone marrow were influenced by intestinal infection driven cytokines. Broadhurst *et al*. (2010) showed that *T. trichiura* infection could induce remission in an individual with ulcerative colitis and associated increase in IL-22^+^ CD4 cells and decrease in IL-17^+^ CD4 cells supporting an immunomodulatory role for human whipworm infection. How *T. muris* or *T. trichiura* achieves such immunomodulation remains to be completely defined but, by analogy with other parasitic nematodes (Hewitson *et al*., 2009) is likely to involve the excretions and secretions (ES) of the worm especially from the later larval and adult stages of the parasite.

Here, we aim to review immunomodulation by *Trichuris* infection with a focus on known and potential immunomodulatory proteins in *T. muris* ES, regulatory RNAs and exosome-like vesicles (ELVs).

### Excretory secretory (ES) proteins and other molecules

The ES of multicellular parasites has been the source of target molecules for disruption of the host/parasite interaction, the hunt for vaccine candidates and a potent source of immunomodulatory proteins. A study in 2018 (Eichenberger *et al*., 2018) provided a detailed characterization of *T. muris* adult ES (day 35 post infection), the *T. muris* secretome. Using two biological replicates 148 proteins were confidently identified corresponding to 34.1% of the total predicted secreted proteins from the *T. muris* genome as based on the presence of a signal peptide. GO pfam analysis suggested a dominance of trypsin peptidases, thioredoxin-like and tetracopeptide repeat molecules and in terms of putative molecular function, a dominance of protein binding, metal ion binding and nucleic acid binding molecules. Proteases are highly represented and a number of serine proteases and whey acidic protein (WAP) domain proteins were also identified. Of particular interest were the SCP/TAPS or CAP-domain proteins which although have been shown to be abundant in other soil transmitted helminths had not been well characterized in clade I nematodes and have been suggested to play multiple roles including potential immunomodulatory function (Cantacessi and Gasser, 2012). Substantial research on the ES of *Trichuris suis* has also been undertaken that has shown multiple and varied immunomodulatory activities *in vitro* for total ES and fractions of *T. suis* ES. This included the suppression of proinflammatory cytokine secretion by activated bone marrow-derived macrophages (BMDMs) and bone marrow-derived dendritic cells, the up-regulation of nitric oxide and arginase and the induction of IL-10 from BMDMs. ES was also shown to suppress the proliferation of CD4+ T cells *in vitro*. Three proteins from ES were identified for further study in recombinant form (a triosephosphate isomerase MW 27 kDa, a nucleoside diphosphate kinase MW 26 kDa and a small nuclear ribonucleoprotein MW 8 kDa) which exhibited modulatory effects on BMDM *in vitro*. Readers are referred to Leroux *et al*. (2018) for more details. A study by Laan *et al*. (2017) demonstrated that *T. suis* ES contained compounds that downregulated inflammatory cytokine production from LPS stimulated human dendritic cells. The active component was identified as prostaglandin E2 (PGE-2), the secretion of which was found to be cyclooxygenase independent. A study by Bancroft and Grencis (unpublished) showed that *T. muris* also secreted a similar molecule, putatively PGE-2, from the L3 stage onwards. Addition of aspirin and indomethacin affected worm motility suggesting that *T. muris* secretion of PGE-2 was similar to that seen in the studies with *T. suis* (Laan *et al*., 2017). Rhoads *et al*. (2000) suggested a chymotrypsin/elastase inhibitor secreted by *T. suis* may act as an immunomodulatory mediator. Adult *T. muris* was also found to have a potential orthologue of l-dopachrome-methyl ester tautomerase (i.e. macrophage inhibition factor, MIF) although little is known as to any possible immunomodulatory activity (Pennock *et al*., 1998). A study by Santos *et al*. (2013) also detected a homologue of MIF in *T. trichiura* extracts using a proteomic approach. This study also identified fructose bisphosphate aldolase and heat shock protein 70 as potential immunomodulatory mediators. Whether any of these molecules play a role *in vivo*, remains to be determined.

The ES of *T. muris* is unusual as although it secretes a mixture of proteins throughout its life in adults 90+% of total secreted protein is a single poly-cysteine and histidine tailed protein termed p43 (Bancroft *et al*., 2019). This protein was shown to have a novel sequence and protein structure but following sub-structure analysis using Probis (Konc and Janezic, 2012) revealed domains with high homology to TSP-1 repeats and IL-13 R*α*-2. It did not show high levels of homology with the cytokine interferon (IFN)-*γ* which had been suggested from previous studies (Grencis and Entwistle, 1997). Structurally p43 is a compact molecule containing 36 cysteines all disulphide bonded, implying a very stable conformation consistent with the harsh environment into which it is secreted, the caecum and proximal colon. It also contains a natural poly-histidine tail which has the potential to bind a number of different divalent metal cations. The observation that domains of the molecule shared homology with IL-13 R*α*-2, a receptor which binds with extremely high affinity to IL-13 neutralizing IL-13 function (Lupardus *et al*., 2010; Karmele *et al*., 2019) raised the possibility that p43 may share a similar function. This would potentially benefit the parasite as IL-13 is known to be critical to expulsion of *T. muris* (Bancroft *et al*., 1998). Indeed, p43 was shown to bind to IL-13 and also glycosaminoglycans (GAGs) putatively through the TSP-1 repeat region with nanomolar affinities. Moreover, IL-13-driven immune responses could be downregulated by p43 both *in vitro* and *in vivo*. p43 is expressed in all larval stages of *T. muris* (Bancroft *et al*., 2019) with the largest quantity of secreted p43 from adult parasites consistent with high levels of production of a poly-cysteine and histidine-tailed protein by other species of whipworm e.g. *T. suis* (Leroux *et al*., 2018). Chronic infection by whipworms is associated with extensive remodelling of the extracellular matrix (Foth *et al*., 2014; Dawson *et al*., 2020), which is decorated with multiple GAGs. Following chronic *T. muris* infection increases in expression of heparan sulphate, fibronectin and collagen I are observed in the infected caecum (Thompson, Sutherland and Grencis, unpublished observations). It is hypothesized that p43 is secreted into the area adjacent to intestinal epithelial cells (IECs) occupied by the parasite and tethers to locally expressed GAGs with high affinity. It is notable that the parasite constantly burrows providing access to both apical and basal epithelium and therefore has access to IL-13 on epithelial cells. Binding analysis is supportive of sequential GAG–IL-13 interactions and the possibility of tethered p43 interacting with IL-13. It is noteworthy that the major effector mechanisms that remove *T. muris* from the intestine, increased epithelial turnover and upregulation of mucins, are driven by IL-13 acting locally in the intestinal niche (Cliffe *et al*., 2005; Hasnain *et al*., 2011). Excitingly, preliminary data reveal that the homologue to p43 in *T. trichiura* also binds to GAGs and human IL-13 and inhibits human IL-13 function *in vitro* (Bancroft *et al.*, unpublished data).

Intriguingly, the interaction of p43 with a model GAG, heparan sulphate, was shown to be highly zinc dependent. The reasons for this are unknown. Zinc is an important metal ion co-factor essential for multiple biological processes. Preliminary research in our laboratory has shown that *T. muris* adult ES contains elevated levels of zinc (Bancroft and Grencis, unpublished). Interestingly, a study investigating the effects of helminth infection showed that it played a clear role in driving serum levels of zinc and iron in infected human populations independent of diet (Lee *et al*., 2019). The mechanisms underlying these observations are likely to be complex and involve parasite, host immunity and the intestinal microbiota as it is known that zinc and iron can influence immune function (Wessels *et al*., 2017) and the microbiota (Knezevic *et al*., 2020). It is tempting to suggest that the dependency of p43 (and the *T. trichiura* homologue) binding activity on zinc indicates that regulation of zinc during infection may be critical in the relationship between host immunity, parasite and microbiota. A role for the poly-histidine tail found in p43 and its homologues in zinc binding remains to be determined.

During natural infection, *T. muris* is acquired by repeated low dose infections. Using a trickle infection approach in the laboratory, Glover *et al*. (2019) showed that there was a steady build up in worm burden coincident with a dominant Th1 response until partial protective immunity was acquired due to the development of a stronger Th2 response. This suggests that adult parasites are present before effective IL-13-mediated immunity is acquired and thus copious p43 is secreted into the intestinal niche, which would bind IL-13 from the developing Th2 response, effectively delaying host protection.

**Fig. 1.**
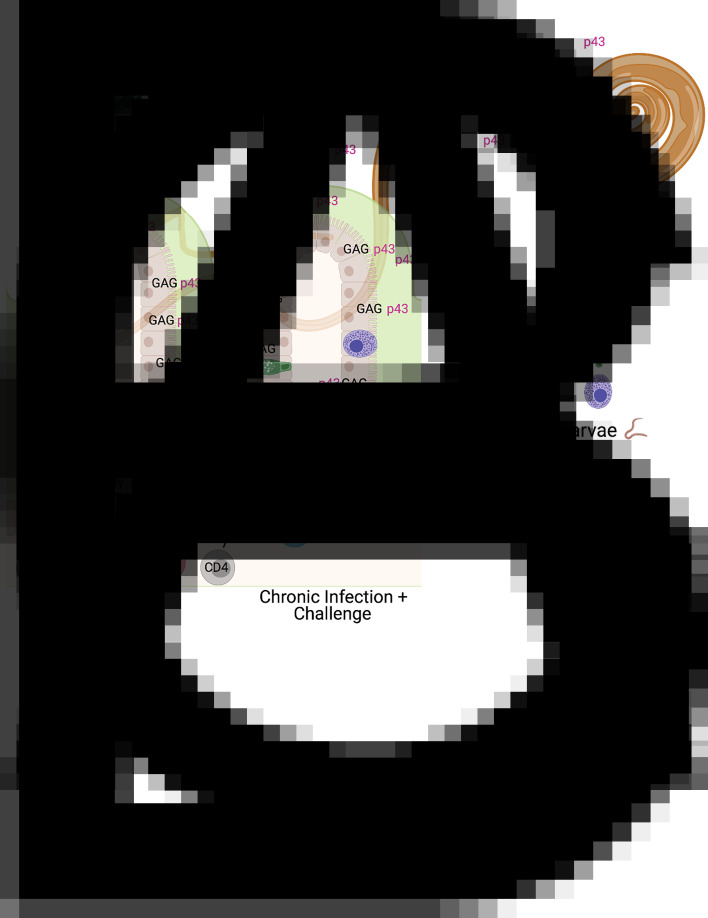
Modulation of protective immunity to *Trichuris muris*. A role for p43 secreted by adult worms through interaction with GAGs and IL-13. (A) Space-filled model of p43 highlighting TSP-1 and IL-13 R*α*-2 homology (red). (B) Space-filled model of p43 docked with IL-13. (C) Diagrammatic representation of caecal epithelium overlaid by thick mucus shown in light green. A single adult worm is shown with anterior end embedded in the epithelium and posterior bulbous end free within the lumen. Chronic infection is IFN-*γ* driven and regulated by CD4^+^ Th cells producing IL-10. Secretion of copious p43 by adult worms is found within the worm niche and binds to extracellular matrix e.g. GAGs with high affinity. Repeated *T. muris* challenge infections following invasion of L1 larvae, as seen in trickle infections, begin to drive the generation of a Th2 response and the production of IL-13. GAG bound p43 will bind IL-13 with high affinity and prevent IL-13 effector function. The net result is a delay and reduced efficacy in host protective immunity, potentially enhancing parasite survival. Models of p43 courtesy of C. Levy.

It is also noteworthy that following a variety of infection regimes little p43 specific antibody is generated and a p43-specific T cell response is largely absent (Bancroft *et al*., 2019). Again, this suggests compartmentalization of p43 to the intestinal niche, which precludes access to the normal antigen processing pathways. However, purified p43 is potently immunogenic when used together with adjuvant to conventionally immunize mice (Bancroft *et al*., 2019). Indeed, to date purified native p43 is the only single *Trichuris* protein that has been shown to be capable of providing almost total protection against challenge infection equivalent to the protection of total adult ES. Importantly, it protects against low dose challenge infection, the type of infection that is more reflective of natural challenge which drives a Th1 response that depresses Th2-mediated protective immunity (Shears *et al*., 2018*b*; Bancroft *et al*., 2019). Although p43 has clear immunomodulatory activity our understanding of potential other important functions for this protein in both parasite biology and host interaction remain to be defined (Fig. 1).

Metabolomic profiling has also been carried out on *T. muris* egg/L1 extracts and adult ES. A study comparing the metabolomes and lipidomes of *T. muris* eggs and *Nippostrongylus brasiliensis* L3 using liquid chromatography-mass spectrometry (LC-MS) showed that many of the major polar metabolites identified have potential anti-inflammatory properties whereas the lipidomic analysis revealed a high level of triglycerides. There was little overlap between these parasites which is perhaps not surprising as they ultimately occupy different niches and have markedly different life cycles (Yeshi *et al*., 2020). Wangchuk *et al*. (2019) used targeted gas chromatography-mass spectrometry and LC-MS of adult ES and identified known polar and non-polar metabolites together with fatty acids including short chain fatty acids (SCFAs). A number of these compounds have been shown to have anti-inflammatory activity in a variety of assays although their activity *in vivo* is unknown. SCFAs that are known to be produced by a number of commensal bacteria and have immunomodulatory effects were also identified although their origin is still a matter of debate. Propionate was the major SCFA detected from *T. muris* although butyrate was also found (Wangchuk *et al*., 2019). Certainly butyrate, which is known to influence colonocyte growth and T regulatory cells, is unlikely to be *T. muris* derived as this species does not contain the genes required to synthesize butyrate or any butyrate transporter orthologues (Foth *et al*., 2014). The source may be related to the fact that *T. muris* has its own intestinal microbiota (White *et al*., 2018).

### Exosomes and exosome-like vesicles

In 2014, Buck *et al*. (2014) showed that exosomes secreted by *Heligmosomoides polygyrus*, which reside in the murine small intestine could transfer small RNAs and nematode proteins to mammalian cells to modulate innate immunity. Tritten *et al*. (2017) isolated ELVs/particles the size of exosomes from adult *T. muris* over an 18–48 h period of *in vitro* culture and from them identified 14 *T. muris* miRNA candidates with high confidence and 73 putative *Trichuris* proteins. Eichenberger *et al*. (2018) identified 364 *T. muris* proteins in ELVs from adult worms, the most common being trypsin domain-protein, sperm-coating protein extracellular proteins, a poly-cysteine and histidine-tailed protein, a glyceraldehyde-3-phosphate dehydrogenase and a TB2/DP1HVA22 domain containing protein. A *T. muris* tetraspanin supportive of ELV formation was also identified. Only 13.7% of the identified *T. muris* proteins had a transmembrane domain and 33% had a signal peptide. Of the 56 miRNAs identified, 34 had homologues to other nematodes and 22 were novel. Shears *et al*. (2018*a*) identified 125 proteins from adult *T. muris* ELVs and again confirmed a number of potential *T. muris*-derived exosome associated proteins many of which did not contain a classical signal peptide. Moreover, the ELVs were able to induce a degree of protection when used for immunization, without adjuvant.

Eichenberger *et al*. (2018) showed that *T. muris* ELVs could be internalized by mouse colonic organoids raising the possibility of the ELVs communicating with intestinal epithelial cells. Duque-Correa *et al*. (2020) developed a novel caecaloid model micro-injected with *T. muris* adult-derived ELVs which enabled study of the host pathogen interaction *in vitro*. Their study argues that the caecum is a unique and different niche to the colon and so provides additional and novel data to that provided in Eichenberger *et al*. (2018). RNA-seq data showed a significant downregulation of viral response associated genes by caecal IECs. A downregulation of IFN-stimulated genes suggested that a direct effect on the caecal epithelium may at least partly explain the anti-inflammatory effects of whipworm infection. This was the first demonstration of an involvement of type I IFNs in *T. muris* infection and Duque-Correra postulated that this immunosuppression may allow ELV cargo entry to the host immune system. White *et al*. (2020) compared small RNAs in ELVs from *T. muris* and *H*eligmosomoides *bakeri* and found they were quite distinct from each other and concluded that this did not depend on their method of preparation but reflected their different intestinal niches and different functions within their hosts. Layton *et al*. (2020) reviewed regulatory RNAs: miRNAs, siRNAs and piRNAs and advocated that miRNAs played a wider role than mere regulation of transcription of the host but also were involved in communication between different taxonomic kingdoms including microbiota.

### Genomic analysis of potential novel immunomodulatory genes

In 2011 the draft genome of *Trichinella spiralis* was published (Mitreva *et al*., 2011) providing valuable information on a clade I parasitic nematode for the first time. Furthermore, in 2014, the publication of the genome of three whipworm species: *T. muris*, *T. trichiura* (Foth *et al*., 2014) and *T. suis* (Jex *et al*., 2014) expanded the available information on clade I nematodes and the potential identification of many genes that could encode immunomodulatory molecules. Collectively, these papers are a wealth of information for studies on host–parasite interactions in whipworm infection. The *T. spiralis* study compared parasitic and non-parasitic genes by evaluating the *T. spiralis* and *Caenorhabditis elegans* genome and also focused on potential vaccine candidates from secreted proteins. The *T. suis* study provided transcriptomic data from different life cycle stages together with the description of small non-coding RNAs. Multiple parasite genes and miRNAs were identified as having potential immunomodulatory activity and the reader is referred to Jex *et al*. (2014) for more details. *Trichuris suis* has been used in trials to treat humans for a range of clinical conditions, most notably inflammatory bowel disease (IBD) (Summers *et al*., 2005), see Hayes and Grencis (2021) supporting an immunomodulatory role for *T. suis* infection.

The *T. muris* reference genome (Foth *et al*., 2014) was the first study to show the pairwise genomes of a major human STH and its murine counterpart. It described both sex and life cycle stage specific genes. It also contained detailed RNA-seq data on host gene expression in chronic low dose *T. muris* infection when immune-mediated regulation is believed to operate. *Trichuris muris* in the mouse has an unusual niche in common with other *Trichuris spp*. with an anterior end forming syncytial tunnels within the intestinal epithelium and occupying between 1000 and 2000 epithelial cells. The large posterior end of the worm hangs free within the caecal lumen. Whipworms also possess atypical structures for nematodes, the bacillary band and the stichosome (Sheffield, 1963) which together occupy the bulk of the anterior half of later larval stages and adult parasites. The stichosome is believed to perform a secretory function associated with digestion whereby stichocytes are ducted into the worm intestine (Lee and Wright, 1978). The bacillary band has been suggested to be involved in both absorption of nutrients and secretion (Tilney *et al*., 2005; Hansen *et al*., 2016; Lopes-Torres *et al*., 2020). Looking at the patterns of parasite gene expression in the anterior region of adult *T. muris*, Foth *et al*. (2014) identified a dominance of chymotrypsin A-like serine proteases and protease inhibitors. These inhibitors had high similarity to a mammalian secretory leucocyte peptidase inhibitor (SLPI). They were encoded for by 63 genes, which is far higher than in any other nematode species so far examined and a high percentage of these genes suggested they encoded secreted proteins based on the presence of a signal peptide. It was suggested that these proteins had potential immunomodulatory function, degrading intestinal mucins which provide a physical barrier to parasite entry into epithelial cells (Foth *et al*., 2014). Certainly, *T. muris* ES contains serine proteases that can degrade the intestinal mucin Muc2 (Hasnain *et al*., 2012). The SLPI-like proteins mostly contained a WAP domain and the *T. muris* genome contained 44 genes which encoded between 1 and 9 WAP domains (Foth *et al*., 2014). WAP domains of unknown function in mammalian SLPIs and elafins have immunomodulatory, anti-inflammatory and anti-microbial properties (Williams *et al*., 2006; Scott *et al*., 2011; Wilkinson *et al*., 2011). They are produced by epithelial cells and have a role in modulating inflammation and wound healing. The direct role of *T. muris*-produced WAP proteins has not been assessed but they have been postulated to play a role in regulating mucosal immunity (Wilkinson *et al*., 2011). Interestingly, one WAP protein from *T. muris* has been used in vaccination studies with some efficacy (Briggs *et al*., 2018). The reference genome for *T. muris* provided a basis for comparison with the human pathogen *T. trichiura* highlighting a number of important similarities supporting a commonality of potential function in human infections (Foth *et al*., 2014).

## Concluding remarks

*Trichuris muris* has been used to inform on the complex interaction between the host, parasite and the rich microbial ecosystem that forms part of its niche. It has also provided valuable information on the host immune system *per se*. Low level infection with *T. muris* has been shown, in immunocompetent mice to result in changes in host pathology and a remodelling of the epithelial matrix. However, considering this parasite breaches the epithelial barrier exposing the host to the caecal microbiota and potential pathobionts, *T. muris* must regulate its environmental niche as many other helminths do. But, it is also evident that the parasite exerts effects beyond its niche. Indeed, infection has been shown to affect the host at sites as far away as the brain implying a systemic regulation. The ES is at the host–parasite interface and is a rich source of molecules and ELVs which have been suggested to have immunoregulatory activity. Publication of the genome of *T. muris* and closely related species has also given scope to uncover previously undiscovered immunoregulatory molecules. What is unusual about *T. muris* (and by extrapolation about other *Trichuris* spp. and clade I nematodes (Giorello *et al*., 2017)), is the dominance of ES from adult parasites quantitatively by a single novel protein, in the case of *T. muris*, p43. This protein has been shown to depress IL-13 function, both *in vitro* and *in vivo*. Excitingly, recent unpublished study shows this also occurs with the homologue of p43 from *T. trichiura* showing potential implications for regulation of clinical outcome in IL-13-mediated pathologies. It is however, also clear that there is a wealth of other *Trichuris*-derived molecules including proteins, small molecules and nucleic acids found in the ES that the parasites potentially utilize to modulate host responses that remain to be characterized and potentially exploited.
